# Supplementary material for: Prevalence and determinants of hypertension among older adults: A comparative analysis of the 6th and 8th national health surveys of Bangladesh
Source: PLoS One. 2023 Oct 16;18(10):e0292989. doi: 10.1371/journal.pone.0292989 (PMC10578599; doi:10.1371/journal.pone.0292989)
Supplement: Attachment — Submitted filename: Response to reviewers.pdf [file pone.0292989.s001.pdf]

Response to reviewers

Reviewer #1: This is a good manuscript. However, 2 points need to be addressed. They are as outlined below:

1) The limitations of this study must be outlined.

2) The utility or uniqueness of this work must be highlighted.

Autor's responses: Thanks for pointed out this issue. Authors are grateful for raising these issues. The limitation and strength have been revised.

Reviewer #2: Prevalence and determinants of hypertension among older adults: A comparative analysis of the 6th and 8th national health surveys of Bangladesh
Manuscript Review:
General:
The rationale is straight forward. The objectives of the study are clear. For the most part the methods are appropriate and well described. The author further summarized the strength and limitation of the study. Despite these strength, there are some aspects of the manuscript that needs to be addressed including several lapses in English construction throughout the manuscript, which makes it difficult to read sometimes. The authors should consider having their manuscript proofread before publication.

Autor's responses: Thanks for your comments. Authors appreciate your comments and have careful revised according to reviewer's suggestions.
Abstract
This is well written and contains all the elements of a good abstract, but there are minor corrections to make:
1. Line 21: add a word "and" in the sentence "… in low-and middle income…"

Autor's responses: Thanks for your comments. Authors have careful revised according to reviewer's suggestions. Modified texts are in red color.

Thank you for your comments. We have carefully revised the manuscript according to your suggestions.

2. The second sentence in background section is not clear, so the author need to rephrase this sentence.

Autor’s responses: Thanks for your comments. Authors have careful revised according to reviewer’s suggestions. Modified texts are in red color.

3. Line 27: add a word “data” for clarity in sentence “……..were assessed in 2011 and 2018 data of Bangladesh….”

Autor’s responses: Thanks for your comments.Authors careful revision has been conducted as per comments. Modified texts are in red color.

4. Line 28: Rephrase the sentence i.e “…enrolled both men and women aged above 34 years and….”

Autor’s responses: Thanks for your comments. Authors have careful revised according to reviewer’s suggestions. Modified texts are in red color.

5. Line 37: The sentence is not clear to the reader, the author need to rephrase “Socioeconomic status (SES) was a major distal determinant of hypertension and had 21% population attributable risk…”

Autor’s responses: Thanks for your comments. Authors have careful revised according to reviewer’s suggestions. Modified texts are in red color.

6. Line 40: The sentence is no clear, please rephrase i.e “……, with 97% of risk mediated by excessive….” Also the author should change the word “aware” to “awareness”

Autor’s responses: Thanks for your comments. Authors have careful revised according to reviewer’s suggestions. Modified texts are in red color.

Introduction:
This is also well written, except for minor corrections
1. Line 58: add a word “and” in the sentence “… in low-and middle income…” similarly to Line 60

Autor’s responses: Thanks for your comments. Authors have careful revised according to reviewer’s suggestions. Modified texts are in red color.
2. Line 93: change “measure of” to “measurement of”

Autor’s responses: Thanks for your comments. Authors have careful revised according to reviewer’s suggestions. Modified texts are in red color.

Methods:

By and large this section is okay, except for minor comments:

1. Some sentences are not clear to the reader and therefore need to be rephrased for the reader to easily follow and understand, for-instance Line 113, 114, 123 and 129:

Autor's responses: Thanks for your comments. Authors have careful revised according to reviewer's suggestions. Modified texts are in red color.

• Line 113: Suggested "of three adults aged more than 34 years"

Autor's responses: Thanks for suggestion. Authors have careful revised according to reviewer's suggestions. Modified texts are in red color.

• Line 114: Suggested "In order to have more focus on non-communicable diseases, the 4th HPNSP 2016-2021 included a component…."

Autor's responses: Thanks for suggestion. Authors have careful revised according to reviewer's suggestions. Modified texts are in red color.

• Line 123: Suggested "The survey involved 18,000 and 20,160 households obtained by using two-stage stratified cluster sampling method in 2011 and 2017-18 respectively"

Autor's responses: Thanks for suggestion. Authors have careful revised according to reviewer's suggestions. Modified texts are in red color.

• Line 129: Suggested "we used data of the 6th and 8th Bangladesh Demographic and Health surveys (BDHSs) conducted from March to October 2011 and from October 2017 to March 2018 respectively"

Autor's responses: Thanks for suggestion. Authors have careful revised according to reviewer's suggestions. Modified texts are in red color.

• Line 237: Suggested "We classified an individual as hypertensive if ...."

Autor's responses: Thanks for suggestion. Authors have careful revised according to reviewer's suggestions. Modified texts are in red color.

2. The author should specify the devices used to measure blood pressure by briefly describing the Model Company and country of origin.

Autor's responses: Thanks for suggestion. Authors have careful revised according to reviewer's suggestions. Modified texts are in red color.

3. The author did not tell how many times Blood pressure was measured and whether was on different occasions as recommended for diagnosis of hypertension. This need to come out clearly.

Autor's responses: Thanks for suggestion. Authors have careful revised according to reviewer's suggestions. Modified texts are in red color.

4. The author should give a brief description of how weight and weight was measured and tools used or can provide a reference of published paper for that work which have these description in the methodology.

Autor's responses: Thanks for suggestion. Authors have careful revised according to reviewer's suggestions. Modified texts are in red color.

5. Author need to provide reference for the BMI cut-off point used.

Autor's responses: Thanks for suggestion. Authors have careful revised according to reviewer's suggestions. Modified texts are in red color.

Results

This section is okay except for minor comments:

1. The author should state clearly the diabetes being referred in this study to be sure is not the other one (Diabetes insipidus vs Diabetes mellitus) i.e Diabetes mellitus (DM)

Autor's responses: Thanks for suggestion. Authors have careful revised according to reviewer's suggestions. Modified texts are in red color.

2. The author should correct grammar error and missing word noted in line 188, 195, 227, 239,

Autor's responses: Authors appreciate your suggestion and Authors have careful revised according to reviewer's suggestions. Modified texts are in red color.

• Line 188: add word "years" suggested "The average age of the study population was 51.3 (SE: 0.17 years)" and SE should be written in long for in the first place with abbreviation in brackets and continue to use abbreviation in subsequent use.

Autor's responses: Thanks for suggestion. Authors have careful revised according to reviewer's suggestions. Modified texts are in red color.

• Line 195: Remove the word "and" after the word "prevalence of"

Autor's responses: Thanks for suggestion. Authors have careful revised according to reviewer's suggestions. Modified texts are in red color.

• Line 227: Suggested to use the word "and" instead of "to"

Autor’s responses: Thanks for suggestion. Authors have careful revised according to reviewer’s suggestions. Modified texts are in red color.

• Line 239: A word “slightly” should be replaced by “slight” and a word “increased” replaced by “increase”

Autor’s responses: Thanks for suggestion. Authors have careful revised according to reviewer’s suggestions. Modified texts are in red color.

• Line 240 – 241: add the word “in” before the word “the 6th survey...”

Autor’s responses: Thanks for suggestion. Authors have careful revised according to reviewer’s suggestions. Modified texts are in red color.

• Line 242: suggested to replace the word “increasing” with “increased”, remove the word “the” before the word “awareness” and before “nutrition” and change the word “improving to “improved”

Autor’s responses: Thanks for suggestion. Authors have careful revised according to reviewer’s suggestions. Modified texts are in red color.

• Line 252: add the word “and” and remove comma between the word “education level” and “excessive body weight”

Autor’s responses: Thanks for suggestion. Authors have careful revised according to reviewer’s suggestions. Modified texts are in red color.

3. Line 191 – 192: I think here the percent should be calculated out of those with excessive body weight but as it stands seems to be calculated out of total. Similarly to line 196 – 197

Autor’s responses: Thanks for suggestion. Authors have careful revised according to reviewer’s suggestions. Modified texts are in red color.

4. Line 192 - 193: The author advised to combine the two sentences. Suggested “The 8th survey in 2018 included 9015 adults with average age of 45.5 (SE: 0.19) for analysis.

Autor’s responses: Thanks for suggestion. Authors have careful revised according to reviewer’s suggestions. Modified texts are in red color.

5. Line 230,234 239 and 242: Add the word “hypertension” before the word “awareness”

Autor’s responses: Thanks for suggestion. Authors have careful revised according to reviewer’s suggestions. Modified texts are in red color.

6. Line 244: The sentence “ The proportion of attributable………” Is not clear. Please rephrase

Autor’s responses: Thanks for suggestion. Authors have careful revised according to reviewer’s suggestions. Modified texts are in red color.

Discussion and conclusion

The discussion and conclusions are by and large in line with the findings. The authors have discussed their results well and the conclusions are appropriate. However, there some minor comments to address for better flow and easy for a reader to follow.

1. The author should correct grammar error and missing word noted in line 261, 268, 273, 286, 300, 309, 312 and 316

Autor's responses: Authors appreciate your suggestion and Authors have careful revised according to reviewer's suggestions. Modified texts are in red color.

• Line 261: replace "growth" with "growing"

Autor's responses: Thanks for suggestion. Authors have careful revised according to reviewer's suggestions. Modified texts are in red color.

• Line 268 and 316: Replace the word "aware" with "awareness"

Autor's responses: Authors appreciate your suggestion and Authors have careful revised according to reviewer's suggestions. Modified texts are in red color.

• Line 273: Add the word "and" between "education level" and "excessive body weight"

Autor's responses: Thanks for suggestion. Authors have careful revised according to reviewer's suggestions. Modified texts are in red color.

• Line 281: Add the word "the" before the word "body"

Autor's responses: Thanks for suggestion. Authors have careful revised according to reviewer's suggestions. Modified texts are in red color.

• Line 286: Replace the word "intake" with consumption

Autor's responses: Thanks for suggestion. Authors have careful revised according to reviewer's suggestions. Modified texts are in red color.

• Line 290: remove "a" before the word strongly

Autor's responses: Thanks. Authors are grateful for raising this issue. Authors have careful revised according to reviewer's suggestions. Modified texts are in red color.

• Line 300: Replace "controlling" with "control"

Autor's responses: Thanks. Authors have careful revised according to reviewer's suggestions. Modified texts are in red color.

• Line 309: Rephrase the sentence

Autor’s responses: Thanks. Authors have careful revised according to reviewer’s suggestions. Modified texts are in red color.

• Line 312: Add the word “and” between words “blood pressure” and anthropometric parameters”

Autor’s responses: Thanks. Authors have careful revised according to reviewer’s suggestions. Modified texts are in red color.

2. Line 271: The author is advised to break this sentence “As a result, the relative risk of unhealthy lifestyle, urban areas and higher SES decreased……..” into two for clarity to the reader.

Autor’s responses: Thanks. Authors have careful revised according to reviewer’s suggestions. Modified texts are in red color.

3. Line 282: The author should give a summary of a link between high blood cholesterol level and development of high blood pressure.

Autor’s responses: Thanks. Authors are grateful for raising this issue. Authors have careful revised according to reviewer’s suggestions. Modified texts are in red color.

4. Line 292 – 293: The author is advised to avoid the use of the word “lack” and instead use the word “poor” or “inadequate”. Also the statement “lack of access to health seeking facilities” is not clear, Please check and correct.

Autor’s responses: Authors appreciate your comment. Authors have careful revised according to reviewer’s suggestions. Modified texts are in red color.

5. Line 294 – 296: The sentence “The most rewarding finding from our study that the upper SES was strong positively…….” Is very long and not clear to the reader. The author is advised to rephrase and break the sentence.

Autor’s responses: Thanks, and appreciate your suggestion. Authors have careful revised according to reviewer’s suggestions. Modified texts are in red color.
